# Recent Improvements in CRISPR-Based Amplification-Free Pathogen Detection

**DOI:** 10.3389/fmicb.2021.751408

**Published:** 2021-09-30

**Authors:** Jian Zhang, Hailong Lv, Linxian Li, Minjie Chen, Dayong Gu, Jin Wang, Yong Xu

**Affiliations:** ^1^Department of Clinical Laboratory, Institute of Translational Medicine, Shenzhen Second People’s Hospital, The First Affiliated Hospital of Shenzhen University, Shenzhen, China; ^2^Shenzhen Institute of Synthetic Biology, Shenzhen Institute of Advanced Technology, Chinese Academy of Sciences, Shenzhen, China; ^3^Guangdong Key Laboratory for Biomedical Measurements and Ultrasound Imaging, Department of Biomedical Engineering, School of Medicine, Shenzhen University, Shenzhen, China; ^4^Guangdong Key Laboratory of Systems Biology and Synthetic Biology for Urogenital Tumors, Shenzhen Second People’s Hospital, The First Affiliated Hospital of Shenzhen University, Shenzhen, China

**Keywords:** CRISPR diagnostics, Cas12, Cas13, pathogen detection, amplification-free, trans-cleavage activity

## Abstract

Molecular diagnostic (MDx) methods directly detect target nucleic acid sequences and are therefore an important approach for precise diagnosis of pathogen infection. In comparison with traditional MDx techniques such as PCR, the recently developed CRISPR-based diagnostic technologies, which employ the single-stranded nucleic acid *trans*-cleavage activities of either Cas12 or Cas13, show merits in both sensitivity and specificity and therefore have great potential in both pathogen detection and beyond. With more and more efforts in improving both the CRISPR *trans*-cleavage efficiencies and the signal detection sensitivities, CRISPR-based direct detection of target nucleic acids without preamplification can be a possibility. Here in this mini-review, we summarize recent research progresses of amplification-free CRISPR-Dx systems and explore the potential changes they will lead to pathogen diagnosis. In addition, discussion of the challenges for both detection sensitivity and cost of the amplification-free systems will also be covered.

## Introduction

Infectious diseases caused deaths account for about a quarter of the global human death, and the rate is higher in the mid-term-income countries (Lozano et al., 2012). Since the beginning of the century, several epidemics have caused great loss of both life and property all over the world (Fournier et al., 2013). Particularly, the outbreak of COVID-19, which is caused by the pathogenic agent SARS-CoV-2 virus, has caused more than 196million infection and 4million death (World Health Organization, 2021) and is right now challenging the public health safety, economic development, and social stability. To effectively stop the transmission and spread of the COVID-19 epidemic, dozens of rapid diagnostic methods, including both the nucleic-acid-based molecular diagnostic (MDx) and immunological methods, have been successfully developed soon after the report of the SARS-CoV-2 genomic sequence. Because of the high accuracy and the ability to distinguish the virus mutants, MDx methods are playing a more and more important role in COVID-19 diagnosis and screening.

Commonly used MDx technologies may include the quantitative polymerase chain reaction technology (qPCR), the isothermal nucleic acid amplification technologies, and the next-generation gene sequencing technology (NGS). Although qPCR is the gold-standard technology for most nucleic-acid-based diagnosis and is well known for its sensitivity, accuracy, and robustness, it requires both expensive instruments and sophisticated technicians to handle the samples and accomplish the reactions (Mahony et al., 2009). Isothermal methods such as LAMP (Loop-mediated isothermal AMPlification) and RPA (Recombinase Polymerase Amplification) have minimal requirements for the equipment and amplify target nucleic acids exponentially at constant temperature; however, these methods usually generate nonspecific amplification products and therefore lead to lower diagnostic specificity (Wang et al., 2015; Phillips et al., 2018). The NGS is good at generating massive data but is limited by its cost, inconvenience, and low speed, which may prevent it from wide application in rapid pathogen diagnosis (Yozwiak et al., 2012; Wilson et al., 2014; Langelier et al., 2018). By contrast, the recently developed CRISPR (Clustered Regularly Interspaced Short Palindromic Repeat) diagnostic technologies (CRISPR-Dx) have shown merits of high sensitivity, specificity, rapidness, convenience, and low cost and have been well recognized as the next-generation diagnostic technology (Chertow, 2018). Moreover, a CRISPR-Dx product was issued with an Emergency Use Authorization (EUA) in COVID-19 diagnosis by the Food and Drug Administration (FDA) in the United States last year, which further demonstrates the great potential of CRISPR technologies in pathogen diagnosis.

CRISPR-Dx mainly employs the *trans*-cleavage activities of certain Cas proteins against single-stranded nucleic acids, which include both DNA-targeting type V Cas12 (Chen et al., 2018; Li et al., 2018a) and RNA-targeting type VI Cas13 (Gootenberg et al., 2017). Upon the recognition and binding of target nucleic acids, Cas12 and the guide RNA form a ternary complex with the target nucleic acids, which is then triggered to unleash the *trans*-cleavage activities and nonspecifically cleaves any single-stranded DNA (ssDNA) sequences in the system. With the employment of this activity, several CRISPR-Dx methods such as HOLMES (Li et al., 2018b) and HOLMESv2 (Li et al., 2019) have been created for target nucleic acids detection. Specifically, at the presence of target nucleic acids, Cas12 is activated to *trans*-cleave the fluorophore quencher (FQ)-labeled ssDNA reporter and illuminate detectable fluorescent signals. Similarly, Cas13 recognizes target RNA and *trans*-cleaves FQ-labeled RNA reporter, on the basis of which methods such as SHERLOCK have been successfully developed (Gootenberg et al., 2017).

To achieve attomolar or higher sensitivity, the CRISPR-Dx step needs to be combined with the target nucleic acid amplification step performed by either PCR or isothermal technologies. The two steps are normally separated but can also be integrated into one tube after careful optimization of the isothermal amplification and *trans*-cleavage conditions (Li et al., 2019; Wang et al., 2019; Joung et al., 2020). However, the amplification procedure not only is a waste of time but also has the risk of aerosol contamination. To overcome this limitation, several research groups have made great efforts in developing amplification-free CRISPR-Dx systems and exploring their application in pathogen diagnosis and beyond (Table 1). In this mini-review, we not only summarize the recent progresses but also discuss about the challenges facing them.

**Table 1:** Amplification-free CRISPR-Dx systems.

Principles	Characteristic	Cas	Targets	LOD (aM)	Time (min)^1^	References
Microvolume	High sensitivity and specificity, complex detection equipment.	Cas13a	miRNA; 16S rRNA; SARS-CoV-2	10	60	Tian et al., 2021
		Cas12a	ASFV; EBV; HBV	29	60	Yue et al., 2021
		Cas13a	SARS-CoV-2	5×10^3^	5	Shinoda et al., 2021
Electrochemical biosensors	High sensitivity and specificity, compact detection equipment, poor reproducibility.	Cas12a	HPV-16; PB-19; TGF-β1	5×10^7^	30	Dai et al., 2019
		Cas12a	N.S.	3×10^7^	60	Zhang et al., 2020
		Cas12a	Dengue virus	10^5^	30	Lee et al., 2021
		Cas12a	HIV-1	10^10^	60	Nouri et al., 2020
		Cas13a	miRNA-19b; miRNA-20a	10^7^	9	Bruch et al., 2019, 2021
		Cas13a	Lung carcinoma related RNA	50	36	Sheng et al., 2021
		Cas9; Cas12a	Parvovirus B19	10^5^; 10^4^	>30	Xu et al., 2020
		Cas9	Human genomic DNA	1.7×10^3^	15	Hajian et al., 2019
		Cas9	Human genomic DNA	N.S.	40	Balderston et al., 2021
		Cas13a	miRNA-17	10^3^	30	Zhou et al., 2020
Cas-based cascade amplification	Compatible with conventional detection platforms, long detection time.	Cas13a & Cas12f	miRNA-17	1.3×10^3^	100	Sha et al., 2021
		Cas13 & Csm6	SARS-CoV-2	50	20	Liu et al., 2021
		Cas12a	HBV; Human bladder cancer–associated single-nucleotide mutation	5	240	Shi et al., 2021
Modified crRNA		Cas12a	SARS-CoV-2; HIV; HCV	N.S.	60	Nguyen et al., 2021
Multiple crRNAs		Cas13a	SARS-CoV-2	1.67×10^2^	30	Fozouni et al., 2021
Metal-enhanced fluorescence	Visible detection, complicated ssDNA probe modification.	Cas12a	Breast cancer gene-1	3.4×10^2^	30	Choi et al., 2021

N.S., not specified.

1 Assay time indicates the approximate incubation time most frequently used in the referred study.

## Research Progresses in Amplification-Free CRISPR-Dx

**Figure 1:**
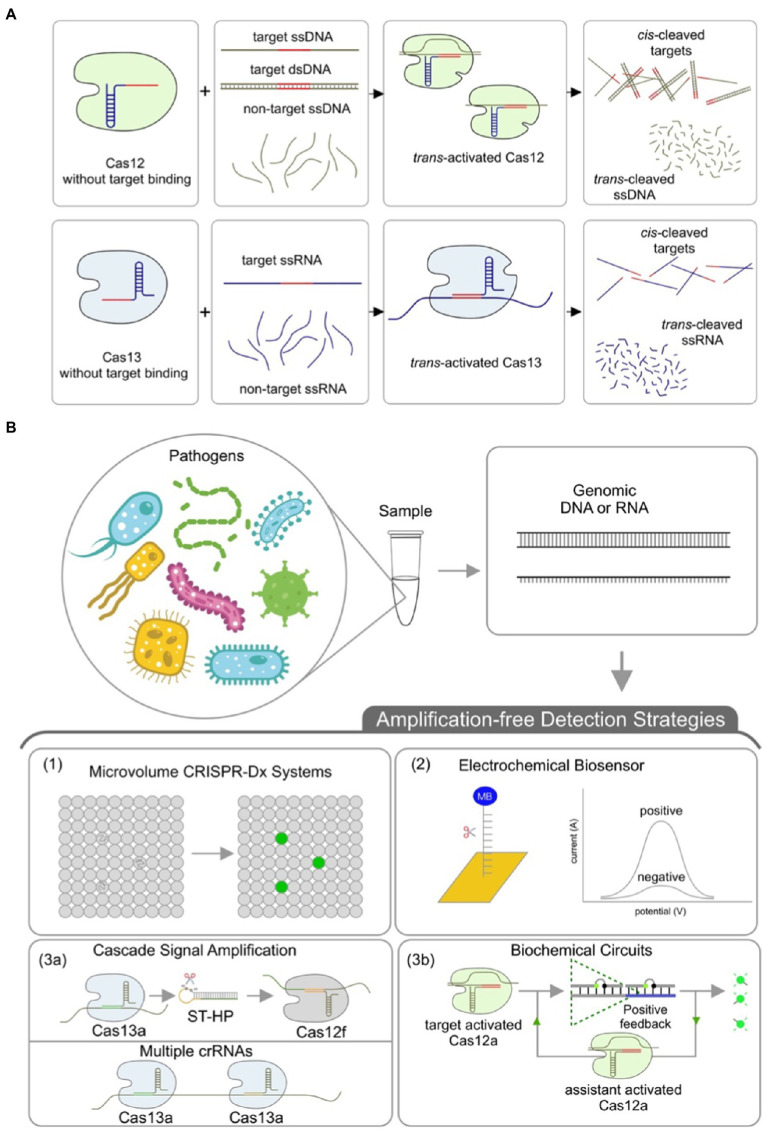
CRISPR-based diagnostics. **(A)** Illustration of both CRISPR *cis*- and *trans*-cleavage reactions. At the presence of target ssDNA or dsDNA, the Cas12/crRNA binary complex specifically binds target DNA and forms a ternary complex, which then both *cis*-cleaves the target DNA and *trans*-cleaves non-target ssDNAs in the reaction system. Similarly, Cas13 recognizes target ssRNA and then forms a ternary complex, which *cis*-cleaves target ssRNA and *trans*-cleaves non-target ssRNAs into small pieces. **(B)** Categories of amplification-free CRISPR-Dx systems. Samples are first treated to release the target DNA or RNA, which is then detected by CRISPR-based diagnostic methods in an amplification-free way. Briefly, the amplification-free CRISPR-Dx methods can be divided into three categories, including (1) increasing the target concentration *via* reducing the reaction volumes, (2) improving the detection sensitivity *via* electrochemical biosensor, and (3) magnifying the output signals *via* either CRISPR cascade amplification (3a) or Cas-mediated positive feedback (3b).

Prior to the Cas-mediated *trans*-cleavage step, the preamplification step increases not only the concentration but also the proportion of the target nucleic acids in the test samples. As the CRISPR technology is of high specificity and distinguishes one base mismatch, the preamplification mainly improves the detection sensitivity of CRISPR-Dx. Therefore, the key factor of developing amplification-free CRISPR-Dx systems lies in the improvement of the detection sensitivity with minimal target nucleic acids. In principle, solutions can be divided into three small categories, including reducing the reaction volume to increase the target concentration, improving the detection sensitivity by electrochemical biosensors, and magnifying the output signals by Cas-mediated cascade amplification or biochemical circuits (Figure 1).

### Microvolume CRISPR-Dx Systems

It has been known that the target concentration is a prerequisite to trigger the *trans*-cleavage activities, e.g., 100 pM DNA for Cas12 (Li et al., 2018b) and ~50 pM RNA for Cas13 (Gootenberg et al., 2017). To develop amplification-free systems, one may simply reduce the reaction volume to increase the target concentration and improve the LOD (limit of detection) without preamplification. For example, a confinement effect is recently introduced to inspire the Cas13a diagnostic system for single-molecule target RNA detection. The target RNA-triggered Cas13a complex is confined in cell-like-sized reactors to increase the local target concentration *via* droplet microfluidics. In comparison with the bulk reaction conditions in microliter volume, the created picoliter-sized Cas13a system demonstrates more than 10,000 times enhancement in detection sensitivity and enables absolute target RNA quantitation (Tian et al., 2021). Using the same strategy, the Cas12a-based amplification-free system is then developed to perform an ultralocalized droplet assay, obtaining single-molecule sensitivity for quantitative target DNA detection (Yue et al., 2021). In a similar way, another group develops a platform called CRISPR-based amplification-free digital RNA detection (SATORI) through combining Cas13 detection and microchamber-array technologies, enabling the detection of target RNA at concentrations as low as 5 fM in 5min (Shinoda et al., 2021).

### Electrochemical Biosensors

The sensitivity of CRIPSR-Dx methods depends on the Cas *trans*-cleavage activities, which are triggered by target nucleic acid to nonspecifically *trans*-cleave single-stranded nucleic acid reporters (Gootenberg et al., 2017; Li et al., 2018a). Usually, reporters are dual labeled with fluorescence and quencher units, and the *trans*-cleavage reactions are monitored by a fluorescence reader. Alternatively, reporters can be labeled with biotin and carboxyfluorescein, and the cleavage is detected by a lateral flow assay (LFA; Kellner et al., 2019). Although the above labeling and detection methods are robust and convenient, the output signals are relatively weak, which may result in limited detection sensitivity. Considering the fact that most target nucleic acids are of a low concentration and the amplification-free CRISPR-Dx systems may generate weak *trans*-cleavage activities, a much more sensitive signal detection approach is required to detect the weak output signal.

Researchers compare various types of biosensing platforms and intelligently combine the CRISPR *trans*-cleavage activities with the electrochemical biosensors, which are highly sensitive, compact, and cheap. Take the E-CRIPSR (CRISPR Cas12a-based electrochemical biosensor), for example, the ssDNA reporter is linked with methylene blue (MB) on a three-electrode-based sensor to monitor the Cas12a *trans*-cleavage activities (Dai et al., 2019). E-CRISPR successfully detects target nucleic acids such as human papillomavirus 16 (HPV-16) and parvovirus B19 (PB-19) at the picomolar level without preamplification of target DNA. The ssDNA reporter can also be optimized to the interfacial hairpin DNA that has an incompact morphological structure to facilitate highly efficient Cas12a cleavage, leading to higher detection sensitivity (Zhang et al., 2020). Using the same electrochemical strategy, Cas12a has been used in direct detection of many other pathogens such as the dengue virus with the LOD of 100 fM (Lee et al., 2021). Besides, the solid-state nanopore sensor has shown great potential in detecting single molecules and is an alternative approach to magnify the signal output. With the combination of Cas12a-mediated *trans*-cleavage and the glass nanopore sensor toward an electronic sensing platform, target nucleic acids such as the HIV-1 DNA can be specifically and sensitively detected without the requirement of preamplification (Nouri et al., 2020).

Cas13a detection can also be combined with electrochemical biosensor through linking the reporter RNA with the glucose oxidase, which produces H_2_O_2_ that can be amperometrically detected, facilitating sensitive diagnosis of miRNAs (Bruch et al., 2019). Through combining the electrochemical biosensor and a dual signal amplification strategy consisting of both Cas13a and a catalytic hairpin DNA circuit, the LOD for target RNAs can be further improved to 50 aM within a readout time of 6min and an overall process time of 36min, without the need of target RNA amplification (Sheng et al., 2021). To detect multiplexed targets, the electrochemical biosensor can be integrated with microfluidic technology by dividing the channel into subsections, allowing for the amplification-free and simultaneous quantification of multiple target RNAs (Bruch et al., 2021).

The above CRISPR-Dx systems rely on both the target-specific binding and nonspecific *trans*-cleavage of single-stranded nucleic acids. Besides, there exist another group of CRISPR-Dx systems that merely employ the Cas-mediated specific binding and *cis*-cleavage and detect the immobilized reporter through electrochemical principle. In the CRISPR-based enhanced electrochemical DNA system (E-DNA), the ssDNA signaling probe first anneals with target ssDNA, forming the double-stranded DNA for subsequent Cas9- or Cas12a-mediated *cis*-cleavage, which removes the electrochemical tag from the probe and induces apparent signal change to facilitate diagnosis (Xu et al., 2020).

Alternatively, through combining catalytically dead Cas9 (dCas9)-based specific binding of target nucleic acids and the graphene-based field-effect transistor, the obtained platform, namely CRISPR-Chip, can rapidly (within 15min) and sensitively (1.7fM) detect unamplified genomic DNA (Hajian et al., 2019), and its improved version termed CRISPR-SNP-Chip demonstrates the ability in detecting single-nucleotide mutations (Balderston et al., 2021). Learning from the principle of the fluorescence *in situ* hybridization (FISH) technology, target nucleic acids were first specifically bound by the complex of dCas9 and sgRNA and then viewed by SYBR Green I (SG I) dyeing, and the obtained dCas9/sgRNA-SG I-based DNA-FISH system has shown advantages in simplicity, precision, and rapidness in detecting pathogens such as the methicillin-resistant *Staphylococcus aureus* (Guk et al., 2017).

### Cas-Mediated Cascades or Biochemical Circuits

Without target amplification, the weak *trans*-cleavage signal can be magnified by Cas-based cascade amplification to facilitate amplification-free diagnosis. Following this principle, Cas13a and Cas12f (previously known as Cas14a) are combined together to develop the casCRISPR system, where the Cas13a *trans*-cleaved products serve as the activators for Cas12f and further trigger the Cas12f-mediated *trans*-cleavage of reporters, illuminating magnified output signals. Noticeably, the casCRISPR system achieves a detection limit of 1.33 fM, i.e., 1,000 times more sensitive than that of Cas13a alone (Sha et al., 2021).

Similarly, the Csm6 RNA endonuclease can also be employed for signal amplification, which was previously employed in the SHERLOCKv2 system to improve the CRISPR detection sensitivity (Gootenberg et al., 2018) and was recently further optimized in the FIND-IT system by using a chemically stabilized activator, facilitating direct diagnosis of target SARS-CoV-2 RNA by Cas13 without preamplification (Liu et al., 2021). Specifically, at the presence of target RNA, Cas13a is triggered to *trans*-cleave a preactivator sequence, generating mature activators for Csm6, which then cleaves the RNA reporter to illuminate fluorescent signals. Moreover, the FIND-IT system can be used for rapid diagnosis of clinical samples, i.e., with a diagnostic sensitivity of RT-qPCR-derived cycle threshold (C_t_) values up to 33 and within 40min of detection time.

The Cas-mediated positive feedback circuit is another excellent idea to amplify the *trans*-cleavage signals. In the CRISPR-Cas-powered catalytic nucleic acid circuit, namely CONAN, there exist two crRNA sequences, pairing to the target nucleic acids and an assistant probe, respectively. The assistant crRNA is blocked by ssDNA FQ-reporter and can be released once the ssDNA blocker is *trans*-cleaved by Cas12a at the presence of target nucleic acids, further guiding Cas12a to target the assistant probe and *trans*-cleave more FQ-reporters in a positive feedback circuit (Shi et al., 2021). Noticeably, the amplification-free CONAN requires only Cas12a for the one-step and real-time detection of genomic DNA with attomolar sensitivity and is so far the most minimalistic of Cas-based signal amplification system.

## Concluding Remarks and Future Perspectives

Under the guidance of CRISPR RNAs, Cas proteins as well as their engineered variants show high specificity in target nucleic acids recognition and binding, which property favors the development of diagnostic methods such as the CRISPR-Chip (Hajian et al., 2019; Balderston et al., 2021) and the paired dCas9 reporter system (Zhang et al., 2017). The subsequent characterization of the *trans*-cleavage activities of Cas12 and Cas13 further improves the detection sensitivity, which has undoubtedly accelerated the development of CRISPR-Dx systems for rapid diagnosis of both pathogens and other target nucleic acids.

Besides, the CRISPR-Dx methods can also be employed as a signal amplifier and combined with approaches such as aptamers and allosteric transcription factors (aTFs) for detection of non-nucleic acid (NNA) targets, which may include metabolic small molecules and metal ion (Liang et al., 2019; Xiong et al., 2020). Usually, the presence of NNA leads to the specific binding of aptamers or aTFs, which then produces specific nucleic acid sequences to trigger the *trans*-cleavage activities of Cas12 or Cas13 to cleaving reporters, illuminating detection signals. Similarly, using an antibody-DNA conjugate, the Cas12a-based Dx was integrated with the enzyme-linked immunosorbent assay (ELISA) to ultra-sensitively detect the pathogen *Cryptosporidium parvum* in a recent study (Li et al., 2021). Although the above CRISPR-based approaches in NNA detection have shown advantages in both detection sensitivities and are amplification-free, we here mainly focused on the most recent progresses of *trans*-cleavage-based MDx technologies, which directly detect target nucleic acids and are of both sensitivity and specificity.

At first, the CRISPR-Dx systems such as SHERLOCK (Gootenberg et al., 2017), HOLMES (Li et al., 2018b), and DETECTR (Chen et al., 2018) consist of two physically separated procedures, i.e., amplification of the target nucleic acids and detection of the amplicons by Cas-mediated *trans*-cleavage. To reduce the possibility of aerosol contamination during transferring the amplified products, scientists subsequently developed one-tube HOLMESv2 CRISPR-Dx system (Li et al., 2019), which combines isothermal LAMP amplification and Cas12b *trans*-cleavage and therefore avoids transferring the amplicons. Albeit one-tube CRISPR-Dx systems may integrate the advantages of both sensitivity and convenience from isothermal amplification technology and specificity from CRISPR technology, the complexity remarkably increases in the optimization of the sequences of amplification primers and guide RNAs as well as the reaction conditions. Moreover, as long as there exists the amplification step, one may still worry about the possibility of aerosol contamination, and more efforts will be required to design complex chips to prevent the contamination event. Therefore, considering both the diagnostic convenience and the cost, it is necessary to develop amplification-free methods, and CRISPR-Dx technologies are so far the most likely to achieve the goal.

Up to now, variant types of amplification-free CRISPR-Dx systems have been successfully developed, and there will be more in the future. Moreover, besides the above-mentioned methods, there exist many other efforts to improve the detection sensitivity. For example, using the principle of metal-enhanced fluorescence, the output fluorescent signals are remarkably magnified by the DNA-functionalized Au nanoparticle gold nanoparticles, which has been demonstrated to support amplification-free detection of target DNA with the Cas12a-mediated *trans*-cleavage activities (Choi et al., 2021). In addition, through optimizing the reaction conditions and designing more than one crRNAs for a target nucleic acid sequence, the detection sensitivity can also be enhanced (Fozouni et al., 2021; Nguyen et al., 2021). Taken together, the present amplification-free methods may use different diagnostic principles and have varied LOD and operational convenience. Albeit some amplification-free CRISPR-Dx methods have demonstrated high detection sensitivity within an acceptable detection time (Hajian et al., 2019; Zhou et al., 2020; Balderston et al., 2021; Sheng et al., 2021; Tian et al., 2021; Yue et al., 2021), the requirement of special chips and instruments as well as the detection cost will limit their wide application and need to be further solved.

In a recent study, Cas13a-based *trans*-cleavage is employed to trigger the exponential amplification of reporter DNAs by both a nicking enzyme and a DNA polymerase. Through further combination of the Cas13a and the electrochemiluminescence technologies, target miRNA can be directly detected at a high sensitivity, e.g., at the LOD of fM level (Zhou et al., 2020). In the future, one can expect that more approaches of distinct principles are integrated to develop an amplification-free CRISPR-Dx method, which is of high sensitivity, high specificity, low cost, rapidness, and good portability.

Without the need of preamplification of target nucleic acids, there will be remarkable reduction of both the detection time and the requirements of the machines and other consumables. Therefore, it is worth expecting that the whole detection can be performed more conveniently with lower cost. Moreover, the application scenarios of CRISPR-Dx can be further explored, e.g., from clinical uses to in-home uses. During the COVID-19 pandemic, there has been a trend toward using home tests for diagnosis of SARS-CoV-2 and several MDx-based products using technologies such as RT-LAMP have been approved by the FDA for more sensitive diagnosis. Compared to the isothermal amplification methods, the CRISPR-Dx results are more specific and there will be no worry about potential risks of aerosol contamination if diagnosed in an amplification-free procedure.

Besides, some modified immunological technologies have also shown their advantages in sensitivity. For example, with the employment of the antibody sandwich protocol for target antigen capture and an ultrabright fluorescence-labeled antibody for signal readout, the obtained method could sensitively detect SARS-CoV-2 and influenza A antigens from clinical samples (Stambaugh et al., 2021). Moreover, some on-chip sample preparation systems could also remarkably promote the hybridization between the capture probes and target virus RNA and enhance the capture efficiency, e.g., through using metered air bubbles to stir up the magnetic beads, which will certainly improve the following detection sensitivity (Du et al., 2017). Therefore, one can expect that different sample preparation and signal detection methods can be integrated with the amplification-free CRISPR-Dx methods to further improve the detection sensitivity in the future. Till then, the amplification-free CRISPR-Dx technologies will find their applications not only in diagnosis of pathogens and genetic diseases but may also in agriculture and beyond.

## Author Contributions

JZ and HL wrote the main manuscript. LL, MC, DG, JW, and YX revised the manuscript. YX and JW coordinated contributions and provided the final draft of the manuscript. All authors contributed to the article and approved the submitted version.

## Funding

This work was supported by grants from the National Natural Science Foundation of China (31922046 and 31770057), Sanming Project of Medicine in Shenzhen (SZSM202011017), the National Key Research and Development Program of China (2018YFA0903700 and 2018YFC0809200), Guangdong Science and Technology Foundation (2020A1515110744, 2020B1111160001, and B2019228), and Shenzhen Science and Technology Foundation (SGLH20180625171602058, 201906133000069, GJHZ20200731095604013, and JCYJ20210317073338001). The sponsors have no involvement in the study design, collection, analysis, and interpretation of data, the writing of the manuscript, and the decision to submit the manuscript for publication.

## Conflict of Interest

The authors declare that the research was conducted in the absence of any commercial or financial relationships that could be construed as a potential conflict of interest.

## Publisher’s Note

All claims expressed in this article are solely those of the authors and do not necessarily represent those of their affiliated organizations, or those of the publisher, the editors and the reviewers. Any product that may be evaluated in this article, or claim that may be made by its manufacturer, is not guaranteed or endorsed by the publisher.
